# Automating Genomic Data Mining via a Sequence-based Matrix Format and Associative Rule Set

**DOI:** 10.1186/1471-2105-6-S2-S2

**Published:** 2005-07-15

**Authors:** Jonathan D Wren, David Johnson, Le Gruenwald

**Affiliations:** 1 Advanced Center for Genome Technology, Department of Botany and Microbiology, 101 David L. Boren Blvd. Rm 2025; 2 School of Computer Science, The University of Oklahoma, Norman, Oklahoma 73019

**Keywords:** Data mining, genomics, format specifications, association rule discovery

## Abstract

There is an enormous amount of information encoded in each genome – enough to create living, responsive and adaptive organisms. Raw sequence data alone is not enough to understand function, mechanisms or interactions. Changes in a single base pair can lead to disease, such as sickle-cell anemia, while some large megabase deletions have no apparent phenotypic effect. Genomic features are varied in their data types and annotation of these features is spread across multiple databases. Herein, we develop a method to automate exploration of genomes by iteratively exploring sequence data for correlations and building upon them. First, to integrate and compare different annotation sources, a sequence matrix (SM) is developed to contain position-dependant information. Second, a classification tree is developed for matrix row types, specifying how each data type is to be treated with respect to other data types for analysis purposes. Third, correlative analyses are developed to analyze features of each matrix row in terms of the other rows, guided by the classification tree as to which analyses are appropriate. A prototype was developed and successful in detecting coinciding genomic features among genes, exons, repetitive elements and CpG islands.

## 1. Introduction

**Figure 1:**
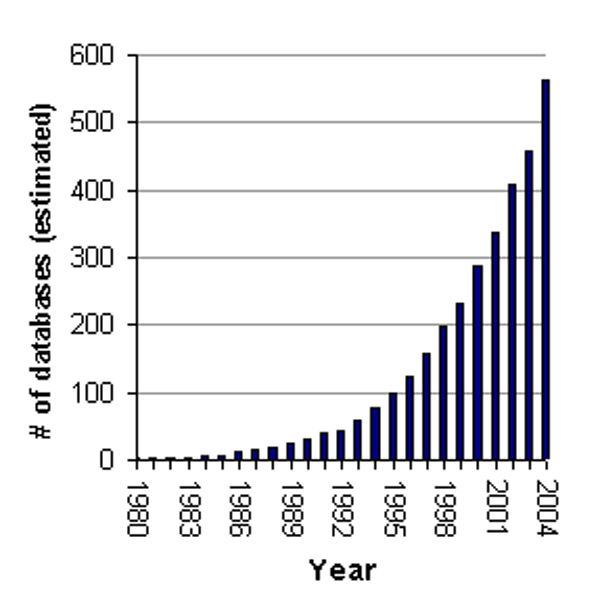
Growth in the number of databases published in MEDLINE by year. Numbers estimated from unique acronym definitions found in MEDLINE ending with the word "database".

As the amount of data gathered and reported in biology and medicine increases exponentially, integration of heterogeneous sources of data becomes an increasingly important part of bioinformatics [1-3]. The number of biomedical databases has been growing rapidly (Figure 1), especially sequence-related databases – storing information on sequence annotation, variation, transcription levels and structural predictions such as protein motifs, families and folds [4]. Many modern technologies are data-intensive and often the rate-limiting step is the ability to derive biological meaning (knowledge) from a series of measurements (data). Data integration is important because a set of data can be used to answer many more questions than it was originally intended to, providing a greater return on the resources invested in gathering the data. Many issues, however, complicate the ability to integrate more than a few sources of data at a time into a central database for analysis. Note that the term "integration" is not intended to simply mean the co-localization of diverse sources of data (e.g. GeneCards [5], a central source for gene-related web links), but a format which permits a single program to draw upon multiple relational data sources for analysis without requiring explicit instructions for handling relations between datasets (e.g., tables).

There are many software packages written for genomic analysis, most of which provide a relatively standard battery of functions: Visualization of sequence features (e.g. repetitive elements, enzyme cut sites, exons), alignment of sequences (e.g. BLAST, BLAT, FASTA), and algorithmic identification of patterns such as genes, promoters and splice sites [6]. The journal *Nucleic Acids Research *publishes an annual issue for databases and web servers, and offers a good overview of what is publicly available. The underlying assumption during the development of most genomic analysis tools is that a user knows – at least to a degree – what he/she is looking for and this may not always be the case. Frequently users are simply looking for what they might describe as "something out of the ordinary". Even packages such as Helix Bioinformatics' GenoStar  that engage in "exploratory genomics", are still driven by user input. Most sequence analysis programs are not designed to accept sets of data as input for analysis (besides phylogenetic packages), such as a series of genes or ontology categories so that common features can be identified. Given the enormous growth in the amount of publicly available data, what is needed is a software package that can systematically examine all available information for statistically significant correlations and report them back to the user.

### 1.1 Data Mining

Data mining is a relatively recent development that has arisen principally as a response to the growing size of datasets, defined loosely as "The nontrivial extraction of implicit, previously unknown, and potentially useful information from data"[7]. In essence, data mining implies that there are answers out there to questions that we have not thought to ask yet – data drives the generation of hypotheses rather than vice versa. As opposed to traditional hypothesis testing designed to verify *a priori *hypotheses about relations between variables, exploratory data analysis (EDA) is used to systematically explore the possibility of relations between variables when there are no (or incomplete) *a priori *expectations as to the nature of those relations. The usage of data-mining techniques in biology and medicine has grown rapidly since 1997, but has always been limited in scope by the number of different features that can be analyzed.

Sequence databases are extremely difficult to "mine" – not only are biological sequences associated with many different data types, but sequence data itself is difficult to fit neatly into any data type. Thus, data mining methods are limited by the amount of information available for analysis within the database they are used upon. The more data that can be integrated into one source, the more potential data mining methodologies have for discovery. Integrating various sources of genomic data into a common format that preserves position-specific information makes it feasible to standardize analysis approaches.

**Figure 2:**
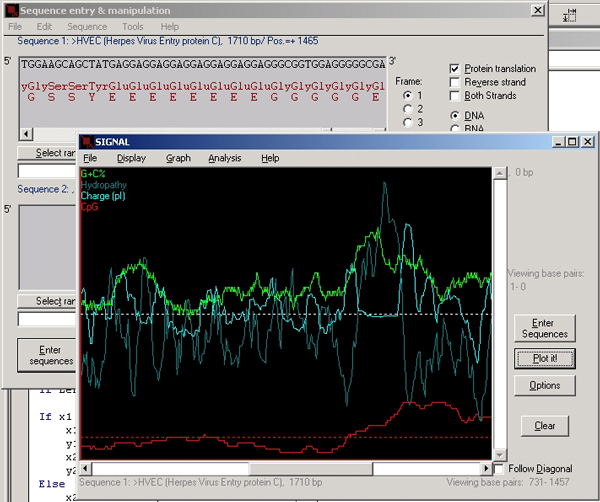
Screenshot of a sequence analysis program, showing a small set of genomic features for the human gene HVEC. In this window the transmembrane domain can be seen (indicated by the hydropathy value peaking while the charge (pI) value falls to zero) next to a CpG island (red line at bottom) and flanked to the right by a polymorphic glutamic acid repeat.

Both data mining and the discovery of new scientific knowledge often proceed through the logical process of induction. Induction entails using specific observations to infer general rules, definitions or categories. For a brief history and discussion of induction through null hypothesis significance testing, see Krueger [8]. In data mining, inductive methods are typically used on well-defined database fields, and are categorical in nature, whereas genomic sequence is vast and varied, with heterogeneous data types. Figure 2, for example, shows a screen shot of a sequence analysis program called SIGNAL (Sequence Information and GeNomic AnaLyses) [9], where a sampling of heterogeneous data types and linkages can be seen. For example, a gene is a single entity but G+C% is calculated from a sequence window inside the gene and is continuous, whereas hydropathy is also continuous but linked to the protein translation instead. When different data types are related to one another in different ways, this makes it difficult to ask general questions (e.g., Do any protein motifs tend to be proximal to polymorphic repeats?) – rather they are answered by constructing specific routines to answer more specific questions (e.g., Do transmembrane domains tend to be proximal to polymorphic repeats?).

As the amount and complexity of data grows, there is an increasing need to be able to ask more open-ended questions, which has been referred to as "data-driven" research as opposed to "hypothesis driven" [10]. It's also accurate to say that the hypothesis driving the research is that informative trends exist within the data and can be found. The goal of this report is to create a "genomic observation system" – one that is able to intelligently employ a set of defined algorithms on a set of genomic fields representing features of interest and then inform the user of what it has found. That is, to note features and/or associations that stand out as statistically significant. However, if the system is truly to be of value in discovering novel associations, it must be able not just to conduct analyses, but to iteratively build upon them. For example, it is interesting to note that some genes have CpG islands upstream and some do not [11], but more interesting is to understand what *types *of genes do or do not have CpG islands upstream and what these genes have in common that would help researchers better understand the significance of CpG islands. CpG islands are stretches of DNA rich in cytosine-guanine pairs, which can be methylated, and can regulate expression of genes. It generally is assumed that CpG islands are involved in modulating "house keeping" genes[12], but recent reports indicate that there are exceptions to this "rule"[13]. To address this example requires at least a two-step process – first the division must be identified, and then genes in each category must be associated or analyzed in some other terms such as function, location, or gene ontology. Because it is not obvious *a priori *which analysis would be best to conduct on what feature to offer insight into any given problem, the value of an exploratory (data mining) approach becomes more apparent.

### 1.2 A data integration format for association rule discovery

There is a strong need in bioinformatics for standardization of analyses and integration of data. At the most basic level is link federation, which is used by web-enabled databases that provide links to other web-enabled databases. Link federation, however, fails to address the issues involved with data integration since it is vulnerable to semantic differences, can fail due to link updates, and shifts the work of integration to the user [14]. Another approach has been view integration, where a wrapper/mediator architecture middleware component is placed between the databases and the user, presenting the data as part of one large system. Examples of this are Kleisli [15], GECKO [16] and AnaBench [17]. These, however, have somewhat failed to take hold due to the need to write a driver for each database to be accessed. View integration also fails to fully address the integration issue as it only brings the data to the user while leaving it up to the user to describe how the data fits together. Additionally, there is the option of data warehousing (e.g., Shah et al [18]), whereby a unified schema is made, data is obtained from heterogeneous sources and then transformed to match that schema. However, this is difficult to maintain, as updates need to be performed, making it vulnerable to frequently changing database schema [14]. It also leaves the relationships between the data up to the user. Finally, there have been attempts to present data in a single type. One such format used has been eXtended Markup Language (XML), using wrappers to obtain the data and LSX to convert it to XML [19]. They have even added support for automated updates to the data [20]. Once the user obtains the data locally, he is left with only one format to read and query. The problem with XML is the same with the other integration options in that it leaves the task of fully integrating data to the user. Each of these means of integrating data has its strengths and weaknesses, but none are well suited for the type of exploratory and adaptive data mining approach we are proposing here.

The idea to develop a modular, standardized exploratory genomic analysis system came from the realization that many questions about genomic properties and correlations involve relatively straightforward correlation analyses, yet software that integrates different data sources is typically written to answer one or a few related questions, usually as a result of individual investigator interest (e.g., see references [21-23]). Data exists in many different types, in many different locations and is produced by many different software packages that run on different platforms. Currently, the number of questions that can be asked is much larger than the number that can be answered, and the more genes, genomes and features that are discovered, the more questions that can be asked. If some method is not developed to begin integrating data and methodologies of examination, then the number of possible questions that can be asked will always exceed our ability to answer them. To bridge this gap, we have developed a prototype for an **automated **(i.e. minimally dependant upon user guidance), extensible, modular and systematic method of searching for correlations within genomic data.

**Figure 3:**
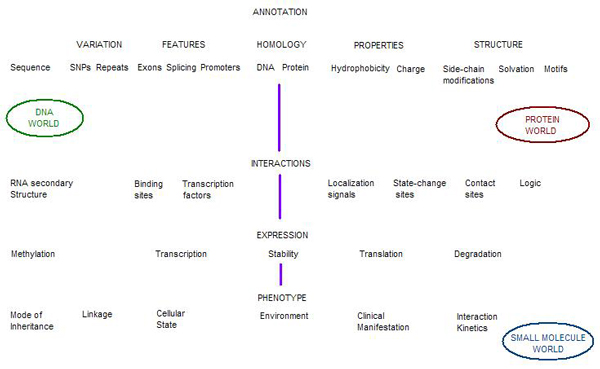
A functional or systems overview of biology. Going from genomic information (top) to creating a living organism with different features/attributes (bottom) involves a number of steps. Evolution begins at the sequence or informational level (left), but the effects of informational alterations manifest at different levels (moving left to right), as DNA is translated into proteins that interact in three dimensions. Molecular biologists obtain information and compile observations at various levels with the goal of linking sequence changes to physical ramifications such as changes in kinetics, solvability or structure. In the end, we want to understand how these alterations in physical properties create phenotypic changes. The ability to integrate sequence-level information with higher-level observations/classifications enables trends to be identified.

To create this automated analysis system, we must first delimit the things we are interested in studying, then we must specify the types of analyses we employ to study relations between, within and among them, and finally we must define the circumstances under which an analysis is appropriate to identify correlations between these things we study. Complicating matters further is the number of sources of data, the heterogeneity of data types, and the functional relationships between data types. Figure 3 offers a graphical overview of biomedically relevant annotation related to genomic sequence.

The approach described herein begins by encoding sequence-based data in the columns of a matrix format that permits position-specific information to be represented, including accommodations made for non-integer data types (e.g. the presence of a gene can be denoted with binary values, but the name of the gene must be linked to an index value). Encoding raw genomic sequence data in the columns of a position-specific matrix permits annotation associated with specific genomic regions to be represented within rows. Annotation includes many data types such as genes, intron-exon boundaries, chemically relevant features such as guanine/cytosine percent (G+C%) content, and gene ontology classifications to name a few. Each row is linked to a row index (RI) that defines the type of values within the row. To enable an exploratory approach, a sequence matrix (SM) classification tree is defined for each data type to be encoded in the rows. This classification tree both limits and guides the types of analyses that are appropriate to perform between rows based upon their type. Finally, to enable the system to build upon previous observations, new rows can be added to the SM when statistically significant correlations are found.

## 2. Construction of a Sequence Matrix Format

Sequence related data can be expressed as a matrix, where rows represent features and columns represent positions in the sequence. The Sequence Matrix format consists of several files. First, each row is expressed as a file. Next, there is an index file that specifies how each file is stored, what it stores, and comparison related information. Programs were written in Visual Basic 6 (SP6) and run on a Pentium 4, 3 GHz machine running Windows 2000 with 1 GB RDRAM. Genomic data was obtained from Genbank  and the August 2004 update was used.

**Figure 4:**
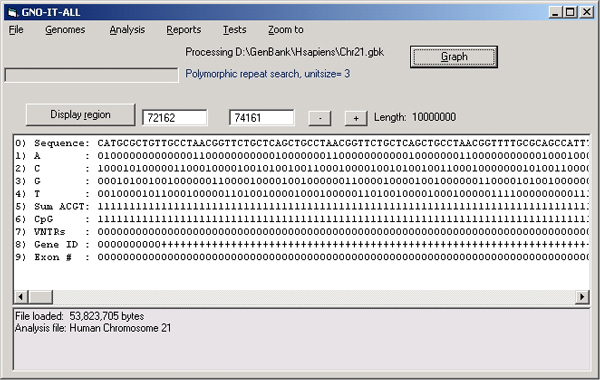
Screenshot of a sequence matrix (SM) display, annotated at the left to indicate what features are being shown in each row. Row zero is not actually part of the SM, but a re-conversion of the numeric matrix into sequence. Sequence variation can easily and accurately be represented in a matrix format. The symbol "+" indicates that the field value is larger than one digit and cannot be shown without visually distorting the display. Here, a CpG island overlaps with a gene's 5' UTR start site.

Figure 4 shows an example of how sequence data is imported into a matrix format. Here, the columns represent position-specific information about a region of genetic sequence. For example, column 1 corresponds to position 72,162 within human chromosome 21. Each of the rows corresponds to different annotations associated with this region. The rows were constructed from Genbank data and include genes, exons and known alternative splice variants. From genomic sequence data, the system automatically calculates G+C% content, CpG islands and periodicity, which consists primarily of Variable Number of Tandem Repeats (VNTR).

Especially of interest is to link genomic features to Gene Ontologies (GO) [24], providing an excellent opportunity to search for functional genomic correlations. Each GO field is encoded with a numeric record ID, permitting it to be easily incorporated into the SM. GO codes have been downloaded and incorporated into a database, each GO category indexed by its ID number.

### 2.1 Developing a classification tree for genomic features

A classification tree of each row type enables an explicit formal specification of how to represent the objects, concepts and other entities that are assumed to exist in some area of interest and the relationships that hold among them. Here, the entities being studied are genomic features and the classification tree being developed is a means of defining how these entities relate to one another and the comparisons that can be conducted between them.

Despite the heterogeneity of data types (e.g. window-based, defined position start and end sites, variable composition), they can be roughly divided into two types: Entity and property (shown below). Entities are defined as portions of the genomic sequence with defined start and end positions that serve a specific function and are either present or not. Introns, for example, are regions that are transcribed into RNA but spliced out in mature mRNA transcripts. Properties, on the other hand, are a range of quantitative or qualitative values that can be taken by a region of sequence. C+G% content, for example, is calculated by examining a window of sequence and varies in its value as the window moves along the sequence. Properties typically have threshold values that delineate genomic features (e.g. C+G% content defines genetic "isochores").

**1.** Entity – A sequence region with a defined beginning and end. Column values can be of type:

•a. Binary – A marker that indicates the existence of an entity, spanning all columns from its beginning to end

•b. Index – A binary-like marker that indicates the existence of an entity, spanning all columns from its beginning to end. The value contained within this field points to an external database unique ID, such as the Genbank Identifier or a Gene Ontology ID number

•c. Range – Possessing a continuous set of possible values

•d. Category – Possessing a discrete set of values, not necessarily sequential

•e. Decimal – Used for entities that are conditionally structured or ordered, such as alternative splice variants

**2.** Property – A genomic feature defined in relation to an entity or region. Column values can be of type:

•a. Binary – Yes or no, on or off (e.g. is a nucleotide within a CpG island?)

•b. Weighted – Frequency of occurrence in relation to another field (e.g. positional variation such as single nucleotide polymorphisms, or possibly exon splice variants)

•c. Range – Possessing a continuous set of possible values (e.g. the number of protein-protein interactions a gene has)

•d. Category – Possessing a discrete set of values, not necessarily sequential (e.g. Gene Ontology IDs)

The goal in development is to keep the classification tree as simple as possible, and as broad as possible on the top node. If a top-level classification tree has only one member, it is probably too specific and not appropriate for inclusion (low-level subcategories can have only one member). Each entry must both make sense and correspond to a specific set or type of analysis to be conducted. Subcategories, similarly, should not lead to entirely different analyses but offer clarification, refinement or exclusion of analyses defined by their parent category.

Some sequence data and annotation has already been obtained from Genbank as mentioned, while a number of different databases are publicly available that provide information for each of the proposed fields in Table 1. The use of matrices to contain sequence data has been well established as a means for analysis of specific, smaller features such as promoters, enhancers and splice sites [25]. The SM proposed is an advanced extension of the Position Weight Matrix (PWM) concept, not limited to weighted values. The SM format permits ID numbers (e.g. database record identifiers) to be used as row values, enabling a quick way of linking position-specific information between databases. There is the possibility of incorporating Unigene EST database into the SM, which may provide a more space-efficient, intuitive representation. EST data permits expression levels to be estimated, along with splice variants and possibly SNP data. However, EST levels are normalized prior to deposition in the database, so inference of transcription levels will be a rough approximation at best and SNP variations might equally be sequencing errors.

### 2.2 Correlative analyses

Induction, defined as attempting to establish a general principle using specific observations, can be used to identify correlations and in data mining is typically conducted via inductive logic programming(ILP) [26]. Induction is limited by the extent to which an induced principle holds true. For example, if all alternatively spliced exons are located within 5 kilobases of an upstream CpG island, this can be induced as a general principle by the system. Note that induction is used to find correlative principles and not strictly defined rules or "truths", because any future alternatively spliced exon in this example that does not have an upstream CpG island would negate it as a strictly defined rule. Induced properties are declared when they pass a thresholded confidence level, and may change upon the addition of new data to the system. The system will automatically cycle through a range of p-values (e.g. p < 0.01 to p < 0.10 stepping in increments of 0.01) to see how performance is affected. That is, to see whether inductions are highly sensitive to small changes in p-values or not. We anticipate they should not be, but if they are then it suggests the possibility that correlations are being formed somewhat spuriously and significance metrics should be reexamined.

Other induction algorithms such as CN2 [27], ID3 which eventually evolved into C4.5[28], and the AQ series [29] to name a few, are not suitable for the proposed study because they are used for different purposes, and require a training set of correct and incorrect inductions to begin learning the underlying patterns before inductive classification can begin. Induction will proceed by correlative analyses since it is not known *a priori *what will be found. An initial, non-exhaustive list of analyses is shown below. Here, a classification tree for analysis defines which analyses are deemed "appropriate" for each data type. Because classification trees are hierarchical, they can be expanded based upon sub-categories to exclude inappropriate analyses. Also, because branches can share parent nodes, it is possible that analyses can overlap between certain sub-categories as appropriate.

#### Analyses

**1**. Proximity – Does the distance between X & Y deviate from random? (e.g. closer or farther?)

**2**. Co-incidence – Are X & Y co-incident with each other more or less frequently than expected?

**3**. Composition – Is there a bias in X for either:

•a. Its frequency/presence within a distribution

•b. Its relative value within a range of values

**4**. Distribution – Does the observed distribution of values deviate from the expected? (e.g. Exon-exon distances). The absolute value of the differences between the areas under the curve (AUC) can answer this question.

**5**. Sequence similarity – e.g. BLAST score (highly CPU intensive when conducting multiple comparisons)

**6**. Gradient – Given a spectrum of values, does the set being analyzed fall within an extreme?

**Figure 5:**
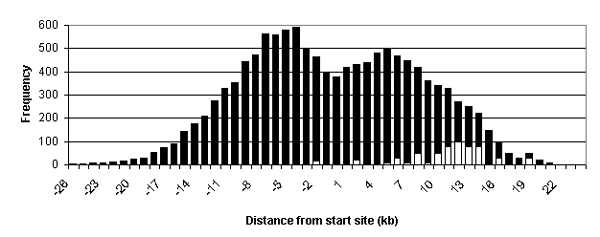
Hypothetical histogram of a binned proximity analysis between genes (entity) and their nearest CpG islands (property), with Gene Ontology categories the subject of analysis. Because the observed distribution is non-symmetrical, bimodal and skewed, statistics that depend upon the central tendency assumption are not appropriate. The black portion of the lines represents the known distribution for all genes while the white portion represents the distribution only for one specific GO category. An MC stochastic simulation tests the null hypothesis that there is no correlation and that the observed distribution could be a result of chance. By picking an equal number of genes as found in the GO category 10,000 times, choosing randomly based upon the observed frequency distribution, and calculating the weighted average each time, we can arrive at a probabilistic estimate of how many times a weighted average equal to or greater than the one observed could arise by chance. Other MC-based statistical tests are possible, such as analyzing the spread of data, but are not explored in this report.

**Figure 6:**
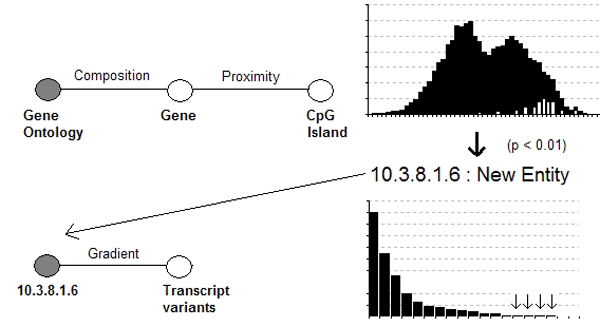
Hypothetical example of how the iterative induction process would work. Extending upon the example offered earlier (Figure 7), a correlation is found by analysis of GO categories – a particular category is statistically overrepresented among genes that have CpG islands located after the start site. It is recorded as a new entity, named after its parent features and analyses that spawned the observation in the first place (10.3.8.1.6 – row #10 connected by analysis #3 with reference to row #8 and compared by analysis #1 to row #6). A new row with a unique name is then created in the SM. This new row is added to the existing SM as the next available number (row 11 in this example), with its name (10.3.8.1.6) providing a means of tracing its origin, and is populated with binary values corresponding to the location of these genomic regions. Later, when the system is re-running correlation analyses, it finds that Row 11 (which was not present during the last analysis run) is correlated with the presence of an unusually large number of transcript variants (arrows at bottom right). The correlation identified is that genes in this GO category also tend to have many splice variants. One might use this correlation to hypothesize that splicing proceeds by "silencing" exons through methylation changes.

**Figure 7:**
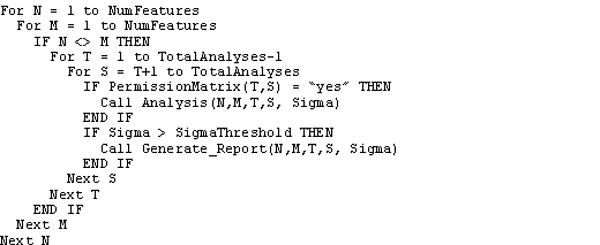
Simplified pseudocode representing the overall logic flow in the program. The user either defines a set of features or all available features are used (NumFeatures). The code then compares each feature to each of the other features, checking for what types of analyses are appropriate. If permission to use a specific analysis on two features is granted, then it is called by passing the matrix rows (N, M) as the subjects of analysis, defines the appropriate analysis (T, S) and returns a value (sigma) representing the significance of correlation between the two.

Application of statistical models for significance analysis (e.g. confidence intervals, p values) depends upon assumptions about the distribution of the data. While it is possible to find appropriate statistical analysis methods for any given genome model (e.g. human), it is not consistent with the goal of the system to be modular and adaptive if users must propose a statistical model for every new genome and genomic feature to be analyzed in terms of every other feature already present. Therefore, we use Monte Carlo (MC) stochastic simulations and bootstrap statistical methods to establish a probabilistic inference for the statistical significance of observations. These methods, while CPU intensive, are less dependant upon assumptions about the distribution of values and have been used frequently in biological analysis [30]. When engaging in an extremely broad comparison of feature versus feature, it's reasonable to assume that distributions will vary and cannot be characterized a priori. An MC correlation testing method can calculate the probability of getting an outcome at least as extreme as the particular outcome observed under the null hypothesis. Figure 5 shows an example of how correlation significance testing would work. When an analysis exceeds a statistical confidence threshold, it will be incorporated into the SM as a row-row correlation. Incorporating this information enables future steps to operate upon it and provides an iterative discovery process (example shown in Figure 6). Figure 7 illustrates the iterative process whereby each row is examined in the context of every other row if an analysis type is permissible.

The addition of new rows to the SM is a critical part of exploratory discovery, yet arguably the most difficult part for several reasons. First, it's necessary to build upon previous observations. Consider an example in which one set of genes has CpG islands upstream in their promoter and the rest do not, CpG islands being necessary for shutting off gene transcription via promoter methylation. Assume, hypothetically, that CpG islands were known as a feature of interest but their function was not known. In theory, the SM data-mining approach should be able to identify that CpG islands are located proximal to the 5' end of genes to a statistically significant degree. This is an observation, yet it is not clear what to make of it without knowing the role of CpG islands. With one additional piece of information, researchers might be able to deduce what this correlation implies and formulate a testable hypothesis for the role of CpG islands. A correlation (negative in this case) might then be identified between genes with CpG islands and an ontological category of basic metabolic processes (i.e. genes that must be expressed in every cell and therefore should not be shut off). Thus, it is necessary to be able to record statistically significant events in some manner, else the algorithm will be nothing more than an exhaustive battery of analyses run against an exhaustive set of fields. The addition of a new row marks the genomic locations of the identified correlation and the *type *of new row added would depend upon the type of correlation identified. In the CpG island example given above, the type of row added would be defined as a set of entities with defined start and end sites (i.e. the genes with CpG islands upstream).

Rather than ask specific questions about genomic correlations, if we can define when an event is statistically noteworthy and select genomic features that might be of potential interest (i.e. we are willing to examine in a report), then we can enable a software program to look for any and all statistically noteworthy associations and report them to us. The system should be able to identify known correlations and, optimally, the system will identify many that have not been documented within the scientific literature. A major pitfall is that the system may overfit the data – that is, it may find strong rules that hold within a given dataset but do not generalize to new datasets. One possible remedy is to warn the user against "statistical" interpretation of significance scores, and to point out significance scores are only used for ranking discovered associations. This is an approach followed in the association rule discovery literature. The traditional machine learning remedy against overfitting is to split data into a training and test set, find correlations on the training set and, for the "best" rules, report significance values computed on the test set. There are also techniques for cross-validation that would be useful for on-the-fly evaluation such as k-fold cross-validation and leave-one-out cross-validation. This type of training is different than the pattern-learning training normally associated with inductive classification – here the training and test sets would be used to define sensitivity thresholds.

### 2.3 Problems of significance

So far we have defined a format and means of automated exploration of row-row correlations, and even a means whereby we hope to circumvent the problems that may be encountered with different data types and distributions, but perhaps the most daunting challenge will be how to establish the significance of a correlation. Using genomic correlations already known to be true, we can first identify minimal significance cutoffs that would miss valid observations were they any more stringent. These cutoffs may well vary by data types compared, which may emerge as the system is developed beyond the current version. Optimal significance cutoffs are more difficult to determine, but the iterative observation procedure outlined in the previous section may enable us to empirically approximate this cutoff. Ultimately, any and all correlations identified by the system can be simplified to the questions "So what?" or "What does it mean?", so we would first expect that the system would not continue to add new observation rows indefinitely. Intuitively, while we expect there may be much that we do not know about the relationship between genomics and biology, there is nonetheless a finite amount. As the system is further developed, we expect to test the growth of new rows, both depth and breadth, and hope this will permit us to adjust significance cutoffs so that infinite matrix expansion is prevented. As biologists examine SM correlations, we would expect that their feedback on the utility/meaningfulness of correlations would enable better adjustment of the system as well in terms of significance cutoffs. That is, if correlations identified by the system have no apparent meaning to the biologist, then perhaps they may be statistically valid, but without biological meaning. For example, two features may coincide with one another at a statistically significant frequency, yet there may be no corresponding implication – it may just be a coincidence. Some correlations may exist in what at first appears to be a vacuum, at least given our current level of understanding, and not make sense until more information is known about the evolution of genomes. Which leads to the final aspect of why we believe the SM approach will be of great utility – examining Figure 8, we see that when a significant correlation is identified it is simply created as a new row. In this example, certain genes with CpG islands located upstream of the start site disproportionately fall within a certain classification tree category. At first, a biologist might concede that this observation seems interesting, but in terms of relevance it falls within the "so what?" category. Later in the iterative analysis, the system might then identify that the positions in this row are strongly correlated with whether or not the first exon is spliced out of a gene. Tracing this row back to its ancestral correlation, it would then be reasonable to hypothesize about the biological relevance of CpG islands located upstream of the start site – perhaps they determine whether or not the first exon is spliced out and offer a possible mechanism (demethylation) by which such an action is possible.

## 3. Prototype testing – finding known correlations *de novo*

A prototype of the described system has been constructed and tested to see if it was able to identify genomic correlations. A routine was written for coincidence analysis – detecting when two sequence features overlap more or less frequently than would be expected by chance. Four fields were tested for linkage: CpG islands, repetitive elements (microsatellites, with 1 to 3 bp repeated sequences), genes and exons. We expected, of course, that genes and exons should be tightly linked and used this as the control. The correlations were run over the first 10 megabases from four different chromosomes: 3, 5, 13 and 18. Monte Carlo (MC) simulations were conducted with each field analyzed, repositioning them randomly within an equal number of base pairs. For the purposes of this analysis it was stipulated that fields did not overlap (e.g. two different genes located within the same contiguous sequence range) – an assumption known to be false in microbes, but holds true most of the time in mammals. Ten MC simulations were conducted for each comparison and the results summarized in Table 2.

Table 2 reveals a number of stronger and weaker correlations and permits an analysis of the range of values to be expected. The control analysis, that genes and exons should always coincide, was compared with random arrangements of genes and exons. The difference in the average number of coincident features observed compared to that observed during MC simulations was 3.02 times higher on average (chromosomes vary regionally in their gene richness). Two other strong correlations stand out, the first is that repetitive elements are relatively uncommon within exons, an observation verified in other studies[31]. The overrepresentation of CpGs in exons but not in genes makes sense given that CpG islands are generally found upstream of genes, including the 5' untranslated region, but genes are generally much larger than their exons (e.g. the first 12 genes on chromosome 6 had over 2.6 MB of sequence compared to only about 46 KB for their corresponding exons). Transcription through C-G base pairings requires separation of 3 hydrogen bonds compared to 2 for A-T. Energy conservation is seen during transcription in that frequently transcribed genes contain shorter exons[32], thus it also makes sense that exons would be more rich than introns in CpGs. Short repetitive elements consisting of 1–2 base pair repeats were far more common in introns than genes[31], so the lack of correlation here also makes sense.

## Discussion

Probably the greatest advantage of SM is that for data analyses of spatially close positions, SM is embarrassingly parallelizable. Data access time can be reduced greatly, by reading windows in parallel, but also windowed comparisons can easily be done in parallel. With Bioinformatic Sequence Markup Language (BSML) data, the data must be pre-processed to know how to divide the data, and the number of chunks it can be broken into while still retaining XML properties limits parallelizing.

Proposed here is a system to enable automated association rule discovery on genomic sequence data. Since genomic data is intrinsically linked to raw sequence data, we have proposed a format by which position-dependant information can be both stored and represented in the form of a sequence matrix where rows represent annotated data linked to the sequence position represented by the column. The explicit linking of row values makes the SM embarrassingly parallelizable, which means it may take advantage of the scaling power that computing clusters have to offer. The sequence matrix enables correlative data mining algorithms to be run, but by itself is static. We have thus developed a means by which data mining can be conducted through the creation of a classification tree for row data types that define how and whether or not one row data type can be analyzed in the context of another row data type. The classification tree enables a means of iteratively examining row-row relations through varying data mining methods. Furthermore, because the classification tree also defines the data type of the correlation between rows, this enables iterative analysis of genomic features by which significant correlations, when observed, can be expanded into new rows. These new rows can then be re-analyzed in the context of other known features, enabling a machine learning system that is able to build upon previous observations. One downside to the SM format is that overlapping features (e.g. genes that share the same coding region on a chromosome) are difficult to model in the current format, whereas markup languages have no problem with this.

In this report, we have demonstrated functionality on a prototype level. Preliminary testing of the system reveals several limitations including, as expected, that the amount of memory on the development computer limits the size of the SM window available for sequence analysis. Currently, about 10 megabases at a time can be efficiently brought into memory for analysis. The conversion of data into a SM format is both memory and CPU-intensive. The workstation used for development does handle the current data load efficiently, likely because the SM currently contains only a dozen or so basic fields. The number of fields simultaneously brought into the matrix for analysis will likely become a rate-limiting step as more fields are added. Ultimately, the system will be more useful when expanded to include:

1. A de novo genome-wide analysis that searches for correlations between all known fields and expands its matrix when statistically significant matches are found.

2. User-defined set-based correlation query that enables a user to select which features they want analyzed within the SM (e.g. a set of genes, chromosomes, or promoter regions) and run the same analysis

3. User-defined incorporation of a new matrix row, requiring them to enter their own matrix values and specify a classification tree structure (or select from existing options). From that point, all currently defined analyses can be performed and reported in terms of existing rows.

Unresolved problems include a means of significance testing for row-row correlations. Herein, we've proposed to use established correlations and Monte Carlo simulations to identify a lower boundary for p-values, but it's not clear yet what the distribution will be in these p-values and whether or not there will be any overlap between the p-values from correlations considered spurious and p-values from known correlations. For example, a weak yet well-established correlation could have a p-value less than 0.2, whereas another correlation that has no apparent biological meaning could have a p-value less than 0.05. It's not clear yet whether this should simply be accepted as a caveat and judgment reserved on the "meaningless" correlation until a later date, or whether p-value boundary refinements will be necessary.

For future directions, there are several functional genomics questions that we are interested in, and hope the system can eventually be used to answer. First, we know that first exons are farther away from second exons and second from third, etc. Do genes that deviate from this trend have anything in common? GO correlations may prove helpful in answering this question. Second, transcriptional regulation by methylated promoters is being increasingly recognized as a possible origin for complex, late-onset diseases. Not all genes have CpG islands upstream, distance from start site to CpG island varies, and so does the number of methylatable sites. Do the extreme values for any of these variables correlate disproportionately with any late-onset diseases? Genes annotated in OMIM titles with the words "late onset", "adult onset" and "variable onset" will be used as an associative data type. As we think to ask new questions that do not fit into any of the defined categories of analysis discussed in this paper (i.e. that would be either a row or analysis), the goal of the SM is to provide a framework to make specific questions more generalizable.

## Authors' contributions

JDW conceived of the ideas presented herein, developed the SM prototype and wrote the manuscript. DKJ helped to run SM analyses under the supervision of LG.

## Figures and Tables

**Table 1:** An initial list of genomic "features" that will be the subjects of analysis by the system.

Features	Classification tree
Gene	Entity
Splice site	Entity, probabilistic
Alu	Entity, probabilistic
Enzyme cut site	Entity, set, categorical
Post-translation Mod	Entity, set, categorical
Protein Motif	Entity, set, categorical
Promoter	Entity, set, categorical
VNTR	Entity, set, categorical
Exon	Entity, set, range
Chromosome	Entity, set, range
Cytoband	Entity, set, range
CpG island	Property (sequence), window-based, binary
G+C% Isochore	Property (sequence), window-based, range
# of interactions	Property (gene), range
Transcript variants	Property (gene), range
Transcription level	Property (gene), range, conditional
Gene Ontology	Property (gene), categorical, hierarchical
Imprinted	Property (CpG Island), categorical (no/maternal/paternal)
Polymorphic	Property (VNTR), range

**Table 2:** Comparing the number of features observed together within the same genomic space against Monte Carlo stochastic placement simulations. The first 10 MB from chromosomes 3, 5, 13 and 18 were used for analysis and the averages shown above.

**Feature Comparison**	**Avg. # of coincident features (c)**	**Avg. # of coincident features in MC simulation**	**Average MC / c**
CpGs vs. Repeats	676	580	1.26 ± 0.35
CpGs vs. Genes	670	550	1.24 ± 0.18
CpGs vs. Exons	167	59	3.08 ± 1.38
Repeats vs. Genes	2698	2600	1.03 ± 0.05
Repeats vs. Exons	43	111	0.38 ± 0.03
Genes vs. Exons	502	171	3.02 ± 0.60

## References

[B1] Venkatesh TV, Harlow HB (2002). Integromics: challenges in data integration. Genome Biol.

[B2] Searls DB (2003). Data integration – connecting the dots. Nat Biotechnol.

[B3] Searls DB (2005). Data integration: challenges for drug discovery. Nat Rev Drug Discov.

[B4] Davies K (2005). The 2005 Database Explosion. Bio-IT World (online).

[B5] Safran M, Solomon I, Shmueli O, Lapidot M, Shen-Orr S, Adato A, Ben-Dor U, Esterman N, Rosen N, Peter I (2002). GeneCards 2002: towards a complete, object-oriented, human gene compendium. Bioinformatics.

[B6] Womble DD (2000). GCG: The Wisconsin Package of sequence analysis programs. Methods Mol Biol.

[B7] Frawley W, Piatetsky-Shapiro G, Matheus C (1992). Knowledge Discovery in Databases: An Overview. AI Magazine.

[B8] Krueger J (2001). Null hypothesis significance testing. On the survival of a flawed method. Am Psychol.

[B9] Wren JD, Mittelman DA, Garner HR (2002). SIGNAL-Sequence Information and GeNomic AnaLysis. Comput Methods Programs Biomed.

[B10] Nakai K, Vert JP (2002). Genome informatics for data-driven biology. Genome Biol.

[B11] Antequera F, Bird A (1993). Number of CpG islands and genes in human and mouse. Proc Natl Acad Sci U S A.

[B12] Gardiner-Garden M, Frommer M (1987). CpG islands in vertebrate genomes. J Mol Biol.

[B13] Costello JF, Plass C (2001). Methylation matters. J Med Genet.

[B14] Stein L (2003). Integrating Biological Databases. Genetics.

[B15] Bajic V, Li J, Ng S, Wong L (2003). From informatics to bioinformatics. Proceedings of the First Asia-Pacific bioinformatics conference on Bioinformatics 2003: February 01, 2003 2003; Adelaide, Australia.

[B16] Theilhaber J, Ulyanov A, Malanthara A, Cole J, Xu D, Nahf R, Heuer M, Brockel C, Bushnell S (2004). GECKO: a complete large-scale gene expression analysis platform. BMC Bioinformatics.

[B17] Badidi E, De Sousa C, Lang BF, Burger G (2003). AnaBench: a Web/CORBA-based workbench for biomolecular sequence analysis. BMC Bioinformatics.

[B18] Shah SP, Huang Y, Xu T, Yuen MM, Ling J, Ouellette BF (2005). Atlas – a data warehouse for integrative bioinformatics. BMC Bioinformatics.

[B19] Shui WM, Wong RK, Graham SC, Lee LK, Church WB (2003). A new approach to protein structure and function analysis using semi-structured databases. Proceedings of the First Asia-Pacific bioinformatics conference on Bioinformatics 2003: February 01, 2003 2003; Adelaide, Australia.

[B20] Shui WM, Wong RK (2003). Application of XML Schema and Active Rules System in Management and Integration of Heterogeneous Biological Data. Proceedings of IEEE International Symposium on BioInformatics and BioEngineering (BIBE): March 10–12, 2003 2003; Bethesda, MD, USA.

[B21] Zhang LV, Wong SL, King OD, Roth FP (2004). Predicting co-complexed protein pairs using genomic and proteomic data integration. BMC Bioinformatics.

[B22] Foissac S, Schiex T (2005). Integrating alternative splicing detection into gene prediction. BMC Bioinformatics.

[B23] Liu Y, Zhao H (2004). A computational approach for ordering signal transduction pathway components from genomics and proteomics Data. BMC Bioinformatics.

[B24] Ashburner M, Ball CA, Blake JA, Botstein D, Butler H, Cherry JM, Davis AP, Dolinski K, Dwight SS, Eppig JT (2000). Gene ontology: tool for the unification of biology. The Gene Ontology Consortium. Nat Genet.

[B25] Claverie JM, Audic S (1996). The statistical significance of nucleotide position-weight matrix matches. Comput Appl Biosci.

[B26] Muggleton S (1992). Inductive Logic Programming.

[B27] Clark P, Niblett T (1989). The CN2 Induction Algorithm. Machine Learning.

[B28] Quinlan JR (1993). C4.5: Programs for Machine Learning:.

[B29] Michalski RS, Michalski RS, Carbonell JG, Mitchell TM (1983). A Theory and Methodology of Inductive Learning: Developing Foundations for Multistrategy Learning. Machine Learning: An Artificial Intelligence Approach.

[B30] Manly BFJ (1997). Randomization, Bootstrap and Monte Carlo Methods in Biology.

[B31] Wren JD, Forgacs E, Fondon JW, Pertsemlidis A, Cheng SY, Gallardo T, Williams RS, Shohet RV, Minna JD, Garner HR (2000). Repeat polymorphisms within gene regions: phenotypic and evolutionary implications. Am J Hum Genet.

[B32] Castillo-Davis CI, Mekhedov SL, Hartl DL, Koonin EV, Kondrashov FA (2002). Selection for short introns in highly expressed genes. Nat Genet.

